# 3D Porous Cu-Composites for Stable Li-Metal Battery Anodes

**DOI:** 10.1021/acsnano.3c02223

**Published:** 2023-07-25

**Authors:** Sul Ki Park, Davor Copic, Tommy Zijian Zhao, Agnieszka Rutkowska, Bo Wen, Kate Sanders, Ruhan He, Hyun-Kyung Kim, Michael De Volder

**Affiliations:** † Department of Engineering, University of Cambridge, Cambridge CB3 0FS, United Kingdom; ‡ Cambridge Graphene Centre, University of Cambridge, 9 JJ Thomson Avenue, Cambridge CB3 0FA, United Kingdom; § Department of Materials Science and Engineering, Kangwon National University, Chuncheon 24341, Korea; ⊥ School of Engineering and Cyber Systems, United States Coast Guard Academy, New London, Connecticut 06320, United States

**Keywords:** lithium metal anode, lithium-ion batteries, 3D porous electrodes, carbon nanotubes, Cu foam

## Abstract

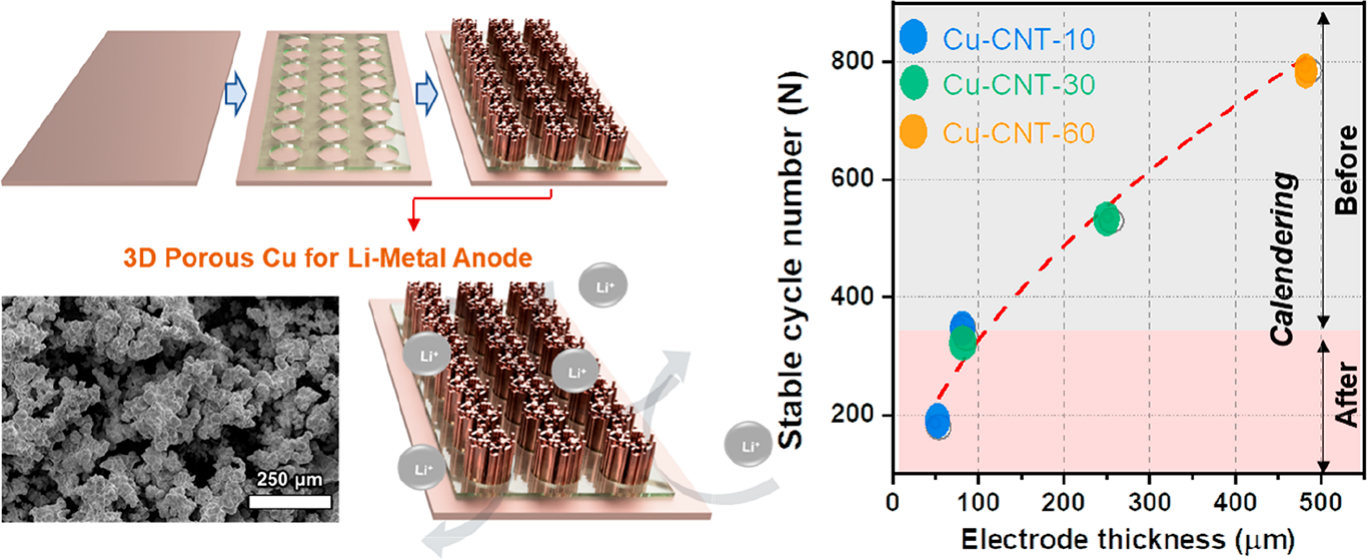

Lithium (Li) metal is a promising anode material for lithium-ion batteries (LIBs) because of its high theoretical specific capacity of 3860 mAh g^–1^ and the low potential of −3.04 V versus the standard hydrogen electrode (SHE). However, these anodes rely on repeated plating and stripping of Li, which leads to consumption of Li inventory and the growth of dendrites that can lead to self-discharge and safety issues. To address these issues, as well as problems related to the volume change of these anodes, a number of different porous conductive scaffolds have been reported to create high surface area electrode on which Li can be plated reliably. While impressive results have been reported in literature, current processes typically rely on either expensive or poorly scalable techniques. Herein, we report a scalable fabrication method to create robust 3D Cu anodes using a one-step electrodeposition process. The areal loading, pore structure, and electrode thickness can be tuned by changing the electrodeposition parameters, and we show how standard mechanical calendering provides a way to further optimize electrode volume, capacity, and cycling stability. Optimized electrodes achieve high Coulombic efficiencies (CEs) of 99% during 800 cycles in half cells at a current density of 0.5 mA cm^–2^ with a total capacity of 0.5 mAh cm^–2^. To the best of our knowledge, this is the highest value ever reported for a host for Li-metal anodes using lithium bis(trifluoromethanesulfonyl)imide LITFSI based electrolyte.

## Introduction

Lithium-ion batteries (LIBs) are the preferred technology for a wide range of consumer electronic devices as well as electric vehicles because of their high energy density.^1−6^ However, there is a constant need for even higher energy densities. One strategy to achieve this is to replace graphite anodes (372 mAh g^–1^ for LiC_6_) with a material with higher capacities. Li metal is promising for this because of its high specific capacity (theoretically 3860 mAh g^–1^) and low redox potential (−3.04 versus SHE).^7−9^ In Li metal anodes, Li is plated on a conductive current collector during charging and stripped during discharging. While this process seems straightforward, it faces several practical challenges: (i) Over time Li dendrites are formed which causes the formation of so-called dead Li ^7,10−14^ or short circuits of the cell.^7,10−14^ (ii) This process leads to excessive solid–electrolyte interface creation, which in turn leads to further consumption of Li inventory. (iii) Li-metal anodes change substantially in volume during cycling, which is problematic from a cell design point of view. These phenomena lead to short cycle life, safety, and other issues which make Li-metal anodes unsuitable for commercialization.^7,10−14^

Impressive research efforts have gone into suppressing Li dendrite formation. One approach is using electrolyte additives to stabilize the solid electrolyte interphase (SEI), such as Cs^+^,^15^ Rb^+^,^15^ boron nitride (BN),^16^ and lithium nitrate (LiNO_3_)^1^ as well as different electrolyte solvent and salts (e.g., LiFSI).^17^ However, obtaining stable SEIs at high areal loadings and high current densities remains a challenge. A second approach to reduce dendrite formation is to control the mechanical pressure applied on the electrodes. Researchers have studied the effect of dendrite growth as a function of the cell stack pressure and balanced the mechanical load taking into account electrolyte infiltration, mechanical stability, etc.^18,19^ A third approach for reducing dendrite growth with three-dimensional (3D) porous anodes provides a higher surface area for the plating process, leading to lower current density per surface area, which reduces the risk of Li dendrite formation. In addition, 3D plating scaffolds reduce issues with volume expansion during plating and stripping. Cu is a logical choice of material for these 3D structures as it is used for anode current collectors in commercial cells because of its high electrical conductivity and electrochemical stability.^2^ Recently, several reports on 3D porous Cu structures with micro-, meso-, and macrosized pores have been proposed using methods such as phase inversion co-tape casting,^20^ multistage heating/washing processing at high temperature,^2^ and the evolution of H_2_ gas which acts as a template.^21^ While impressive, these techniques are complex and challenging to scale-up. Therefore, it is crucial to develop alternative facile and scalable technologies for the design of 3D porous Cu as Li-metal anodes.

In this work, we demonstrate a scalable electrodeposition method to create Cu foams with suitable pore structures for Li metal plating and stripping. Electroplating of Cu is known to create a range of different porous structures;^22,23^ however, these are usually brittle and readily break off the substrate (see picture in Supporting Information). Here we show that by co-plating carbon nanotubes (CNTs) with Cu, a mechanically resilient structure is made that can easily be assembled in coin cells or even calendered without signs of brittle fracture. As deposited, these 3D Cu-CNT composites have an open pore-structure with Cu coated over the CNTs. The latter is important as it means that the CNTs are not creating any excessive surface area that could lead to further SEI formation.^24,25^ In addition, the height of the 3D Cu-CNT composites proposed in this paper can be controlled by simply varying the electrodeposition conditions. Furthermore, this process inherently allows for scale-up manufacture. For instance, in this work, we fabricated batches of up to ∼40 coin cell electrodes in parallel. We studied the deposition of Li metal on these structures with operando optical cells and optimized the electrode density using calendering. The proposed 3D structures exhibit outstanding cycle life with high CEs of 99% for up to 800 cycles in a half-cell configuration. To the best of our knowledge, this result is the highest value ever reported for a host for a Li-metal anode in an LITFSI based electrolyte.

## Results and Discussion

### Preparation and Li Plating/Stripping of 3D Cu-CNT Composite

**Figure 1:**
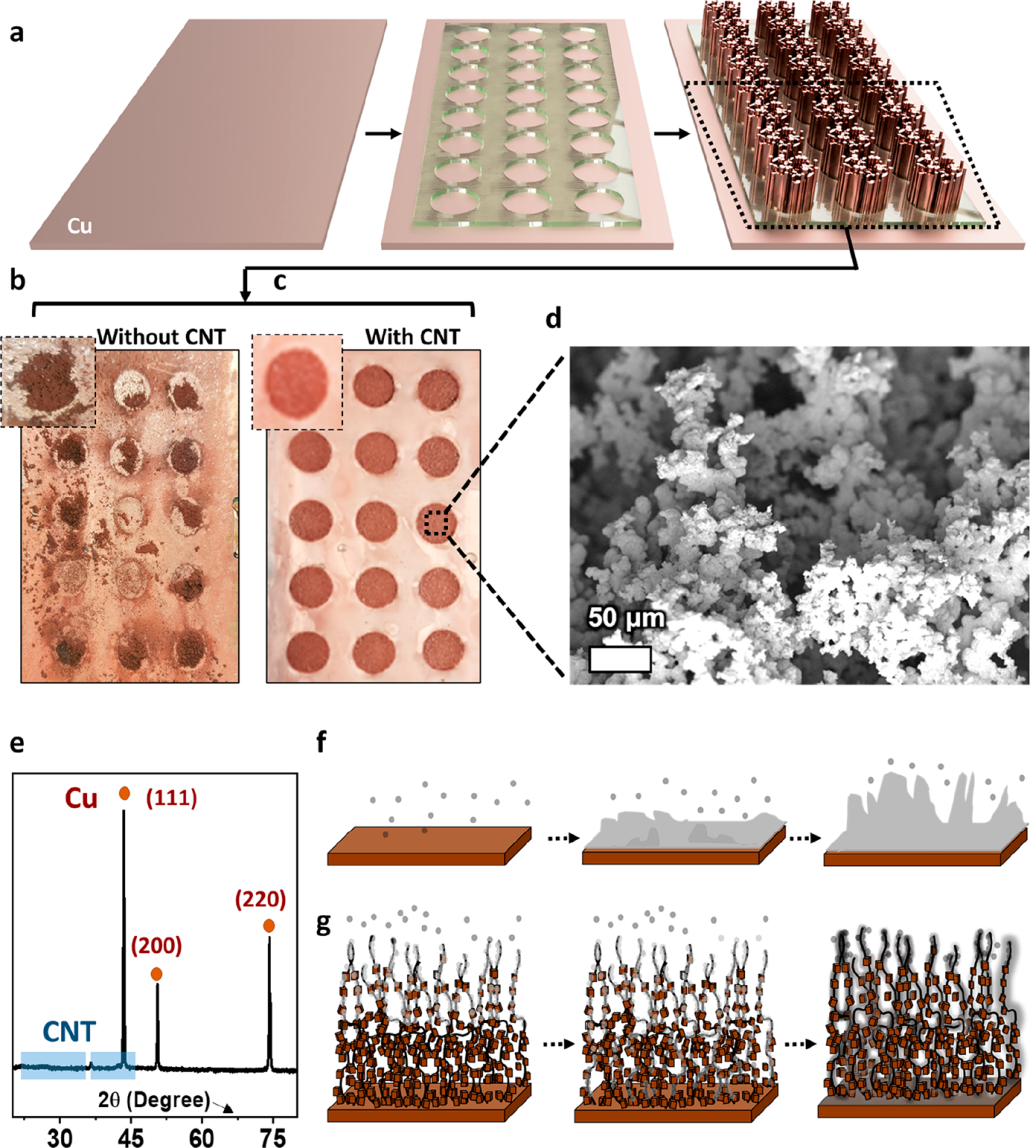
(a) Scheme of the electroplating process for fabricating porous 3D Cu-CNT. Photos of the as-fabricated structure by the electrodeposition process (b) without and (c) with CNTs. (d) SEM images of as-fabricated 3D porous Cu-CNT after electrodeposition. (e) XRD pattern of the 3D porous Cu-CNT composite. Schematic illustration of Li plating/stripping of (f) a bare Cu foil and (g) a 3D porous Cu-CNT composite.

The electroplating process for fabricating porous 3D Cu-CNT composites is shown in Figure 1a. A Cu foil is used as the electrode substrate and an aqueous CuSO_4_ electrolyte solution (0.5 M) in which oxidized multiwall CNTs (Nanocyl NC7000) are dispersed (0.1 wt %) by ultrasonication. The plating process is carried out with a simple two-electrode system using a constant current set to 1.2 A cm^–2^. This process results in open pore-structures shown in Figure S1. Furthermore, we found that by masking the Cu foil with a patterned polymeric foil containing 12 mm diameter holes, circular electrodes can be made to size that directly fit coin cells (Figures 1a–c and S2). The importance of adding CNTs to the Cu composites was further tested by electroplating 3D Cu dendrites without CNTs. The resulting structure is very different, as shown in cross-sectional SEM images (Figure S3). Importantly, Cu foams without CNTs were found to be extremely brittle (Figure 1b,c), and therefore they are difficult to handle and show worse electrochemical performance. Electrochemical impedance spectra (EIS) show that both the series resistance (*R*_s_) and charge transfer resistance (*R*_CT_) decrease substantially when adding CNTs to the foam. This is again an indication of the better integrity and material properties of the CNT-Cu composites (Figure S4).

Figure 1d shows an SEM image of a 3D porous Cu-CNT composite after electrodeposition. To the best of our abilities, we were unable to distinguish any CNTs in these images, suggesting that they are entirely embedded in the Cu material. The presence of CNTs in the Cu matrix is confirmed by XRD measurements (Figures 1e and S5). The reflections for all the 3D porous Cu-CNTs at 2θ = 43.45°, 50.58°, and 74.25° (Cu Kα) can be indexed to the (111), (200), and (220) peaks of cubic Cu phase (JCPDS 04-0836), respectively (Figure 1e).^26^ In addition, the XRD patterns exhibit diffraction peaks at 2θ = 25.66° and 42.53° matching the (002) and (100) reflections of the graphitic structure of CNT, respectively (Figure S5).^24^ As shown in Figures S6 and S7, these results show the presence of Cu, O, and C from the Cu source in the 3D Cu-CNT composite.

As illustrated in Figure 1f ,g, we anticipate that the large surface area of our porous electrodes offers more sites for Li plating^20,21^ and improves the reversibility of the plating process compared to flat Cu substrates depicted in Figure 1f (this is verified experimentally later on).^20,21^

### Morphology Changes of 3D Cu-CNT Composite during Li Plating/Stripping

**Figure 2:**
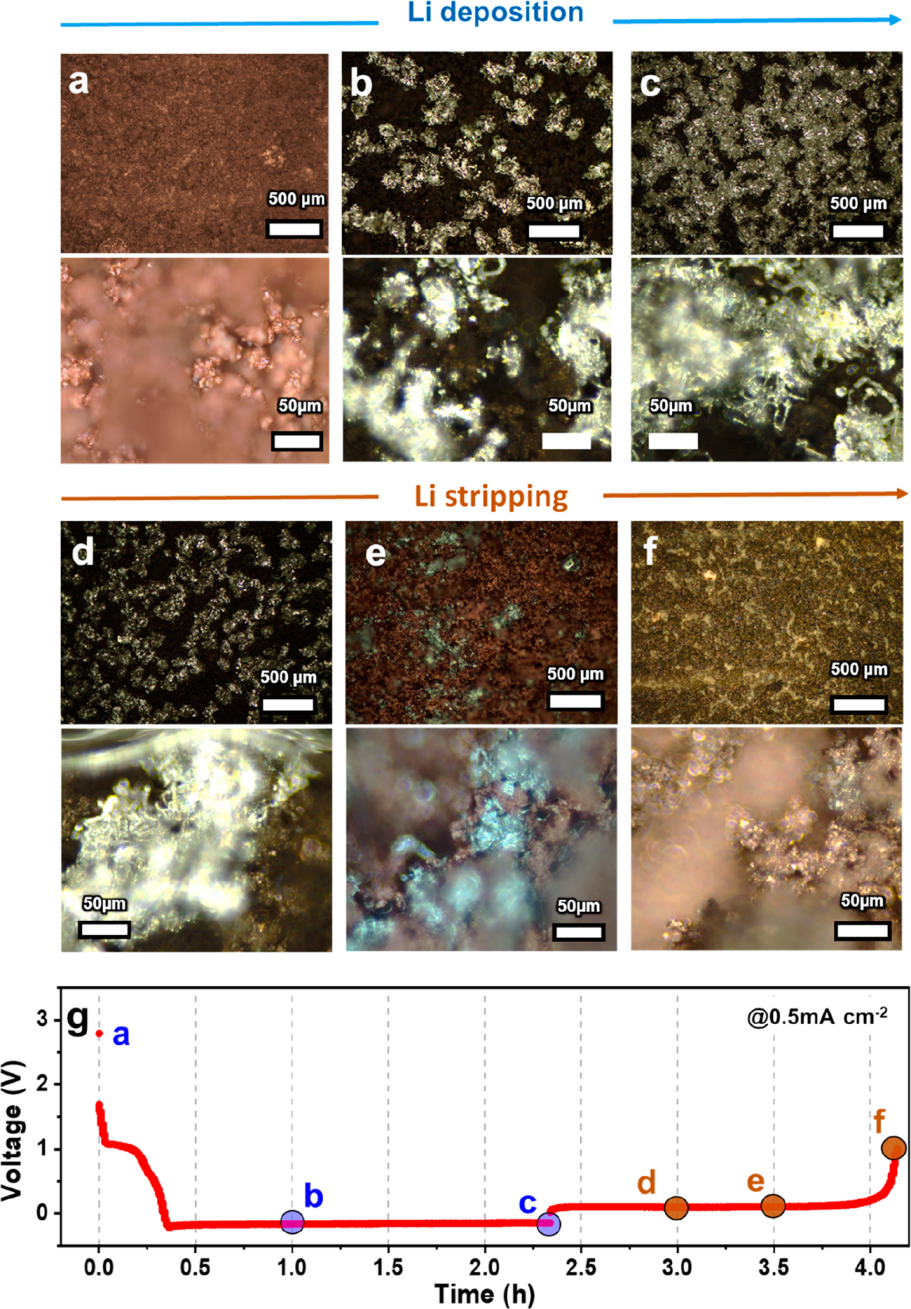
Morphology of Li-metal anode during the depositing/stripping process. Dark field optical microscope images of (a) pristine 3D Cu-CNT without Li metal and depositing (b) 0.50 mAh cm^–2^, (c) 1.17 mAh cm^–2^ onto the 3D Cu-CNT. Anodes after stripping (d) 0.84 mAh cm^–2^, (e) 0.59 mAh cm^–2^, and (f) 0.27 mAh cm^–2^ (recharged to 1 V) from the composite Li-metal anodes (0.9 mAh cm^–2^) with 3D Cu-CNT are indicated in (g) galvanostatic discharge/charge voltage profile at the current density of 0.5 mA cm^–2^.

We subsequently analyzed the performance of these 3D Cu electrodes as anodes for Li plating in battery applications. All electrochemical tests were carried out in CR2032 coin cells using 1 M LITFSI in DOL/DME (1:1) with 3 wt % LiNO_3_ as the electrolyte. We first investigated the morphology of the plated Li metal on the proposed Cu-CNT foam, using optical microscopy to image the electrodes at different Li plating/stripping stages (Figure 2). This was achieved by disassembling electrodes at different states of charge and mounting them in an optical battery cell (EL-Cell) inside a glovebox. After plating 0.50 mAh cm^–2^ of Li onto the 3D Cu-CNT anode at a current density of 0.5 mA cm^–2^, the Li metal seems to cover the surface of the Cu-CNT anode uniformly (see color change in Figure 2b). After depositing 1.17 mAh cm^–2^ of Li, needle-shaped Li protrusions are observed (see close-up in Figure 2c). After stripping Li back to 0.84 mAh cm^–2^, some dendrites seemed to be removed. Finally, at the end of the stripping process (1 V vs Li), a few silver signs of (dead) Li deposits are visible (Figure 2f), which is essential to maintain a good Coulombic efficiency (CE). In previous publications, it was suggested that local defects on Cu can lead to local Li plating, and this in turn can lead to Li dendrite growth (Figure 1f).^20,21^ It seems that the intricate microstructure of our electrodes offers a lot of Li nucleation sites, possibly due to the exposed grain boundaries. This would explain the observed uniform Li coverage from the early stages of lithiation (see Figure 1g).

### Morphology and Cycle Stability for Li Plating/Stripping of 3D Cu-CNT as the Electrodeposition Time

**Figure 3:**
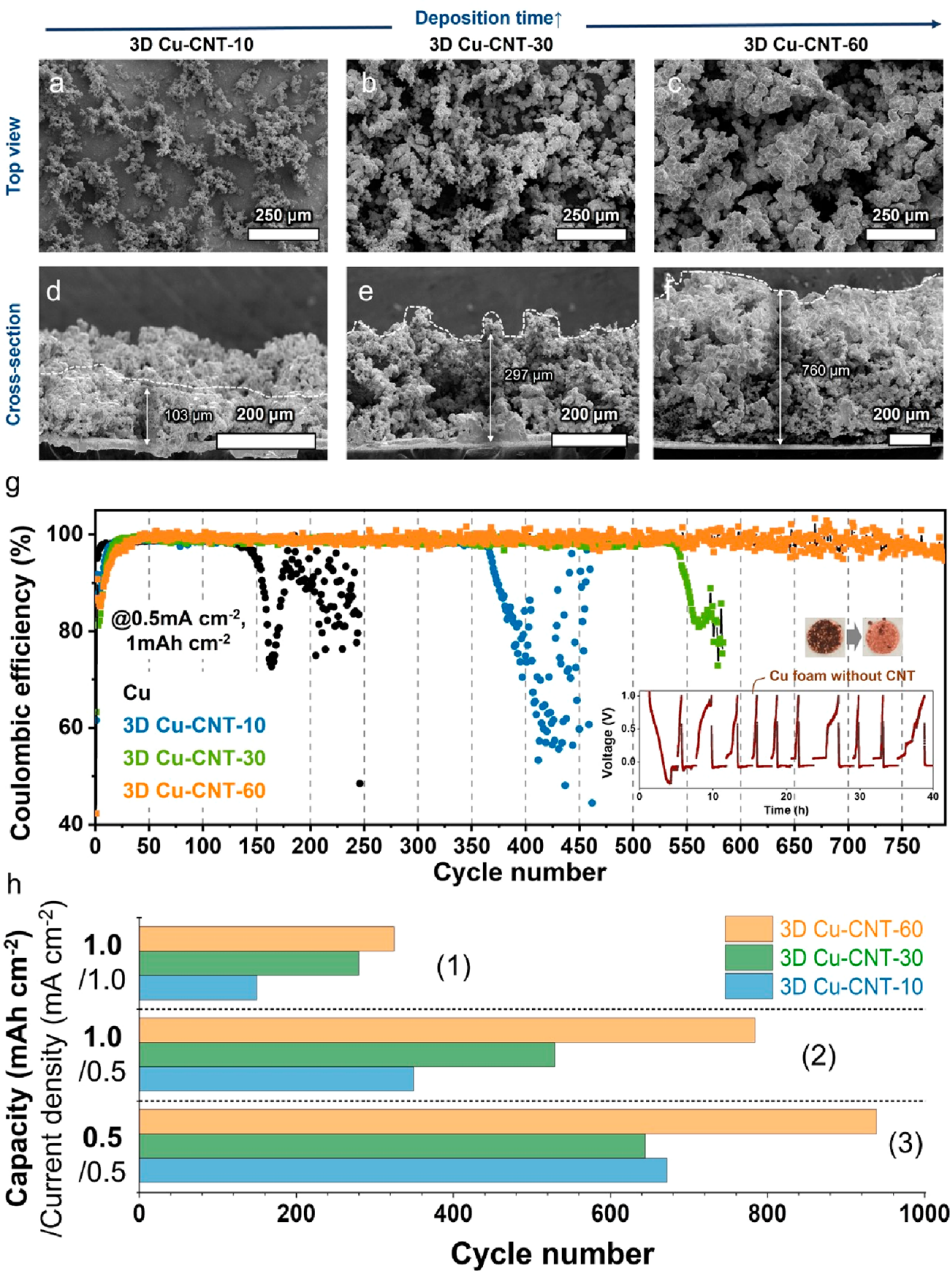
SEM images of (a–c) the top and (d–f) cross-section views of 3D Cu-CNT-10, -30, and -60 (3D Cu-CNT-10, -30 and -60 are named as the electrodeposition time 10, 30 and 60 min, respectively). (g) CE obtained from Li//Cu and Li//3D-Cu-CNT-10, -30, and -60 at a current density of 0.5 mA cm^–2^ with a total capacity of 1 mAh cm^–2^ (inset: a picture and voltage profile of the brittle Cu foam by electrodeposition without CNT). (h) Stable Li CE of 98% obtained from Li/3D-Cu-CNT-10, -30, and -60 at various current densities and total capacities.

The thickness (and mass) of the deposited Cu foam can be varied by controlling the electrodeposition time (Table S1). For instance, prolonging the electrodeposition time from 10 to 30 and 60 min increases the mass loading of Cu foam from 20.2 to 40.6 and 99.0 g cm^–2^ and the height from 103 to 297 and 760 μm, respectively (see Figure 3d–f). The porosity of these samples is studied with the Barrett–Joyner–Halenda (BJH) and Brunauer–Emmett–Teller (BET) methods. For 10 min plated samples, we found a surface area of 3.4 m^2^ g^–1^ and density of 0.01 cm^3^ g^–1^ and a height of 82 μm; by increasing the deposition time to 60 min, the height of the porous Cu increases to 486 μm and the porosity decreased (these heights were measured using a micrometer) (Table S1). We assume that the latter result is because over time, the electrodeposition process increases the thickness of the foam structure, reducing the surface per mass ratio.

Next, we measured the CE in half cells over repeated cycling. Figure 3g and Figure S9 show the CE of the 3D porous Cu-CNT (3D Cu-CNT-10, -30 and -60 are named as the electrodeposition time 10, 30 and 60 min, respectively) and flat Cu foil as a reference at different current densities of 0.5 and 1 mA cm^–2^ and a total transferred capacity of 0.5 and 1.0 mAh cm^–2^. At a current density of 0.5 mA cm^–2^ and a capacity of 1 mAh cm^–2^, the initial CEs of cells with bare Cu foil, the 3D Cu-CNT-10, -30, and -60 are 96.2, 61.6, 63.2, and 42.3%, respectively. The 3D Cu-CNT electrodes show a lower CE compared with bare Cu foil, which might be due to their larger surface area and therefore larger amount of SEI formation on the first cycle. On the other hand, the increased surface area reduces the local current densities and therefore the tendency for dendrite and dead Li formation. As a result, the CEs of the proposed 3D Cu structures increase to 98–99% in only a few cycles and we observe stable cycling for up to 1000 cycles (see further). The control experiment using bare Cu foil shows obvious, drastic fluctuations in CE after only 135 cycles at a current density of 0.5 mA cm^–2^ and 79 cycles at 1 mA cm^–2^ (total capacity of 1 mAh cm^–2^), which is an indication of cell failure (Figures 3g and S9).

Further, to confirm the importance of adding CNTs to the Cu foam, the electrochemical performance of Cu foam obtained by electrodeposition without CNTs was also measured. As discussed above, the Cu particles grown onto Cu substrates are brittle and fracture readily during electrode preparation (see Figure 1b and Figure 3g, inset). These brittle Cu foams yield poor electrochemical cycling stability, as shown in Figure 3g, inset, which further confirms the importance of using 3D porous Cu-CNT composites with well-distributed current densities for Li plating and electrochemical stability without swelling and deformation.

Next, the effect of changing the current densities (0.5, 1, and 2 mA cm^–2^) and capacities (0.5, 1, 2, and 4 mAh cm^–2^) on the cycle life (Figure 3h) and CE (Figure S10) was studied. As shown in Figure 3h and Figure S10, both higher current densities and areal capacities reduce the lifetime as expected for a metal anode system. On the other hand, the lower current densities and capacities lead to longer cycle life as expected. Importantly, these data also show that the thickest electrodes (Cu-CNT-60) possess the highest cycle stability, which is in agreement with our assumption that higher surface area electrodes are beneficial.^1,2,9,21,27^ In addition, as the capacity increases from 0.5 to 1.0 mAh cm^–2^ with the same current density of 0.5 mA cm^–2^, the difference in the cycling stability between 3D Cu-CNT composites is greater (see (2) and (3) in Figure 3h). The average CE of as-fabricated 3D Cu-CNT-60 is 98.3% up to around 950 cycles, which gradually decreases to an average CE of 98% after 1000 cycles (0.5 mA cm^–2^ and 0.5 mA h cm^–2^) (Figure S9b). To the best of our knowledge, our Cu-CNT-60 collector electrodes have a better average CE performance compared to other reports using Cu, carbon and nickel substrates for Li deposition in 1 M LITFSI based electrolyte.^1,2,21,28−31^

### Structure and Cycle Stability for Li Plating/Stripping of Calendered 3D Cu-CNT Composites

**Figure 4:**
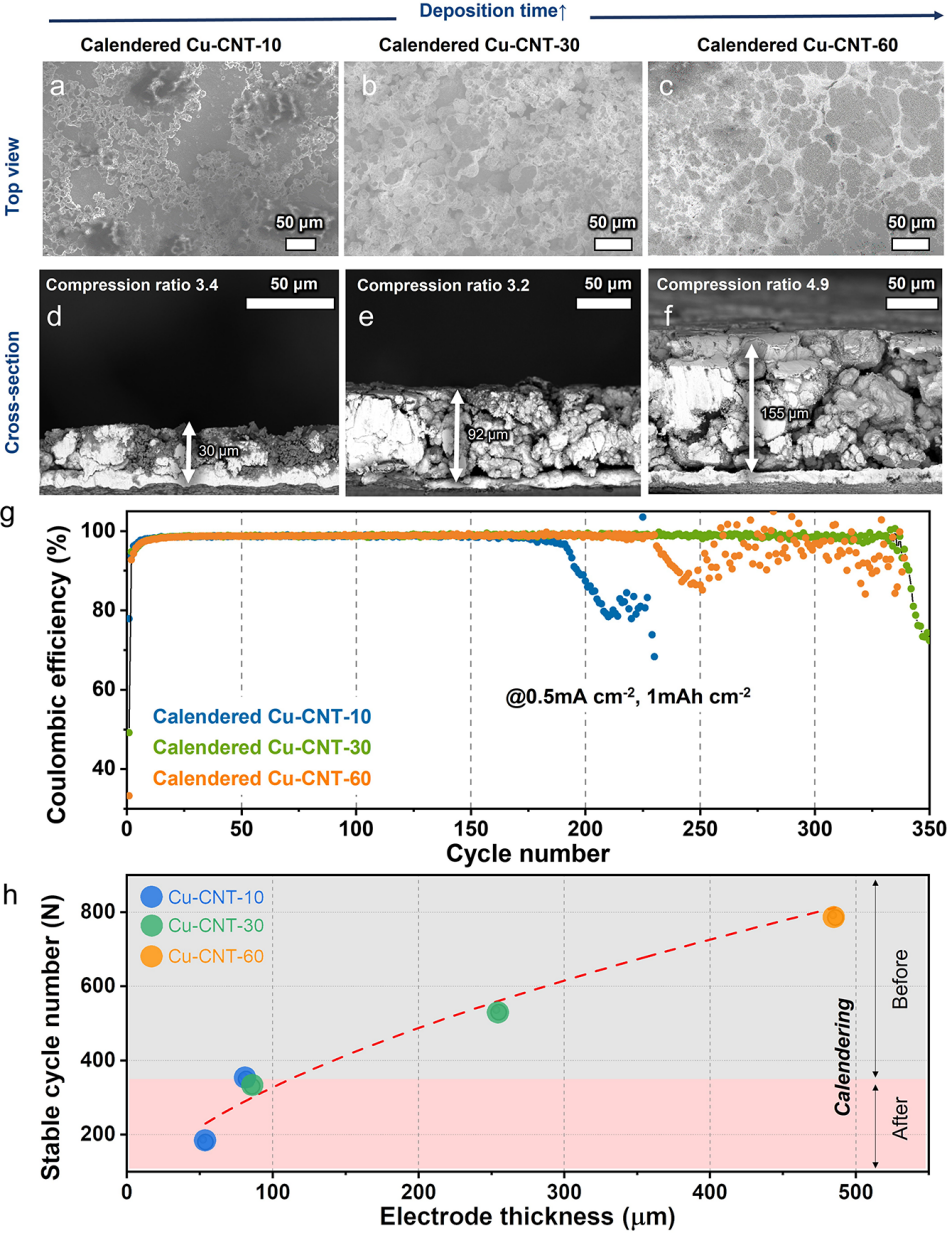
SEM images of (a–c) the top and (d–f) cross-section views of calendered Cu-CNT-10, -30, and -60 after calendering. (g) CE obtained from Li//Cu and Li//calendered Cu-CNT at a current density of 0.5 mA cm^–2^ with a total capacity of 1 mAh cm^–2^. (h) Stable cycle number (CE > 98%) as a function of the electrode thickness for Cu-CNT-10, -30, and -60 electrodes before and after calendering (note that the 60 min samples were overcalendered).

Because of the mechanical stability of our Cu-CNT composites, they can be calendered with the foam deforming plastically rather than fracturing, which was observed for Cu foams without CNT additives. Mechanically calendering the electrodes offers a method to further control the electrode porosity and therefore to balance the electrode volume, capacity, and cycling stability. Figure 4a–c and Figure 4d–f show SEM images of a calendered Cu foam sample (top view and cross section, respectively). Compared with as synthesized 3D Cu-CNT, calendered Cu-CNT-10, -30, and -60 samples show a lower porosity. The calendering process reduced the thickness of Cu-CNT-10, -30, and -60 samples from 103, 297, and 760 μm to 30, 92, and 155 μm, respectively. The Li deposition/stripping on calendered 3D Cu-CNT was conducted at a current density of 0.5 mA cm^–2^ and 1 mAh cm^–2^ (Figure 4g). When comparing the CEs before and after calendering, the calendered Cu-CNT-10, -30, and -60 electrodes have a lower cycling stability with the CE dropping to 98% after 180, 330, and 230 cycles, respectively, after calendering compared to 350, 530, and 785 cycles prior to calendering. This decrease in stability is due to the collapse of the pore structure and reduction in surface area as they are compressed to a ratio of 3.2–4.9. Interestingly, when comparing the as synthesized 10 min and calendered 30 min samples, both have a similar thickness of ∼100 μm and achieve a similar cycling stability of ∼340 cycles. The cycling performance is directly linked to the surface area of the electrodes, and excessive calendering of the electrodes is clearly detrimental. This is exemplified by the 60 min samples, which we calendered to a ratio of ∼4.9 instead of 3.2, resulting in a decrease in cycling stability from 785 to 230. Figure 4h and Table S2 show the cycle number at which the CE drops below 98% for different amounts of Li plating and stripping before and after calendering. Overall, the proposed foam fabrication and calendering processes allow for tuning the thickness and porosity to suit the needs of the battery application.

### Cycle Stability for Li Plating/Stripping of Symmetrical Cells

**Figure 5:**
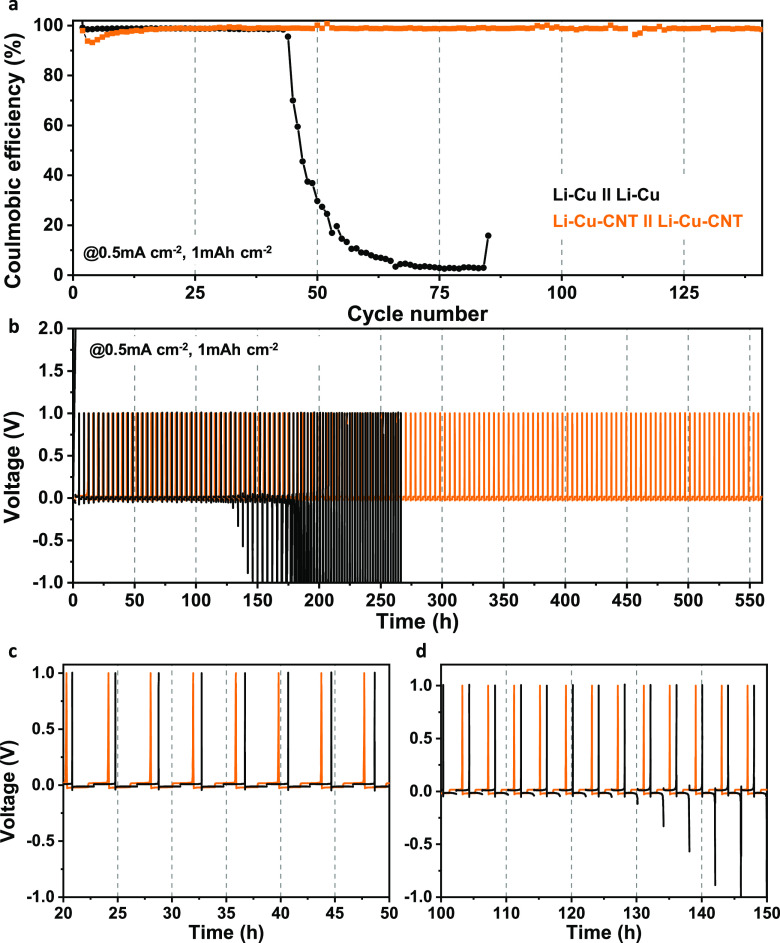
(a) CE and (b, c) voltage time profiles for cycling performance in symmetrical Li-Cu//Li-Cu and Li-Cu-CNT//Li-Cu-CNT at a current density of 0.5 mA cm^–2^ with a capacity of 1 mAh cm^–2^.

To further investigate the practicality of our anodes, the galvanostatic cycling performance of symmetrical Li-Cu-CNT//Li-Cu-CNT cells was measured (Figure 5). Before cycling, 5 mAh cm^–2^ of Li was deposited on the current collector in each half cell at 0.5 mA cm^–2^. These symmetric cells were cycled at a current density of 0.5 mA g^–1^ with a fixed capacity of 1 mAh g^–1^ at 1 mA cm^–2^. These Li-Cu-CNT//Li- Cu-CNT cells display a very stable cycling performance up to 140 cycles (550 h, Figure 5). In contrast, the CE of reference electrodes using flat Cu sheets (Li-Cu//Li-Cu) decreases rapidly after 44 cycles (140 h) followed by a gradual growth in plating overpotential. Finally, our 3D Cu-CNT anodes were tested in a full cell with lithium iron phosphate (LiFePO_4_, LFP) cathodes and the same electrolyte as in previous experiments. As shown in Figure S11, we obtained a capacity retention of 73% after 20 cycles, without any further optimization of the electrodes.

## Conclusion

This work presents a scalable manufacturing process for the fabrication of porous Cu foams for Li-metal anodes. These foams can be tuned in thickness and porosity by adapting the synthesis conditions and calendering. The Li plating and stripping process was first studied using optical microscopy, revealing uniform plating and stripping. This is confirmed by galvanostatic cycling experiments showing 3D Cu-CNT-60 electrodes with stable CEs of up to around 950 cycles at 0.5 mA cm^–2^ with a total capacity of 0.5 mAh cm^–2^, which to the best of our knowledge is a record for the class of materials and LiTFSI electrolyte used in this study. In addition, the 3D Cu-CNT anode//LFP full cell also showed good capacity retention of 73% after 20 cycles.

## Methods

### Fabrication of 3D Porous Cu-CNT Anode

The 3D porous Cu-CNTs are fabricated by electrodeposition. A two-electrode setup was used, in which the working electrode was a clean Cu foil, and the counter electrode was a Cu plate. The electrolyte was a 0.5 M copper sulfate solution in sulfuric acid and a 0.1 wt % CNT dispersion of 2 mL dissolved in DI water. The 3D porous Cu-CNT is prepared by electrodeposition at 1.2 A cm^–2^ for 10, 30, and 60 min in the electrolyte. The as-synthesized 3D porous Cu-CNTs were dried at 60 °C overnight.

### Electrochemical Measurement

CR 2032 coin cells were assembled for all electrochemical measurements in an Ar-filled glovebox. The electrolyte was 1 M LITFSI (lithium bis(trifluoromethanesulfonyl)imide) in DOL (1,3-dioxolane)/DME (1,2-dimethoxyethane) (1:1 by volume) with 3 wt % LiNO_3_.

To evaluate the CE, half cells with 3D porous Cu-CNT and Li metal as the working and counter electrode, respectively, were assembled. Microporous polyethylene (Celgard) was used as the separator. The diameter of the 3D porous Cu-CNT electrodes and the separator is 12 mm and 19 mm, respectively. The assembled cells were cycled between 0 and 1 V (versus Li^+^/Li) at a current density of 0.5 and 1 mA cm^–2^ using LAND cycler (Wuhan Land Electronic Co., Ltd.). A fixed amount of Li (1 mAh cm^–2^) was deposited onto the current collector, and then the Li was stripped in each cycle.

For the long-term cycling test, symmetric cells with Cu foil or the 3D porous Cu-CNT as the working electrode and Li foil as the counter electrode were fabricated. Before cycling, 5 mAh cm^–2^ of Li was plated on the current collector at 1 mA cm^–2^. Then the cells were cycled at different current densities of 0.5 mA cm^–2^ with a plating-stripping capacity of 1 mAh cm^–2^.

For the full cell test, LFP and as-fabricated 3D Cu-CNT were used as the cathode and anode, respectively. LFP, Super P and PVDF (8:1:1) were mixed in NMP and then pasted on Al foil and then dried at 120 °C under vacuum for 12 h. The full cell was cycled at potential from 2.4 to 4.0 V at 0.2 C in 1 M LITFSI in DOL/DME (1:1) with 3 wt % LiNO_3_ using Biologic VMP3.

### Characterizations

Morphologies of electrode materials were observed by scanning electron microscopy (SEM, a Leo variable pressure instrument with an acceleration voltage of 10 kV). X-ray diffraction (XRD) patterns were obtained by using a Bruker D8 Advance instrument (Cu Kα radiation, 6° min^–1^ scan). Nitrogen physisorption was obtained by using a Micromeritics TriFlex porosimeter. X-ray photoelectron spectroscopy (XPS) data were measured by Thermo Scientific K Alpha^+^. Energy dispersive X-ray spectroscopy (EDS) was measured by JSM-7900F.

## Supporting Information Available

Schematic illustration of 3D porous Cu-CNT composite, pictures of the Cu substrate before and after the electrodeposition, SEM images of a Cu foam after the electrodeposition without CNTs, EIS of Cu foam and 3D Cu-CNT, XRD pattern of 3D Cu-CNT, XPS spectra of 3D Cu-CNT, EDS mapping of 3D Cu-CNT, BET adsorption–desorption isotherm graph of 3D Cu-CNT, the areal loading and the information on porous structure for 3D Cu-CNT, CE obtained from 3D Cu-CNT at a current density of 1 and 0.5 mA cm^–2^ with a total capacity of 1 and 0.5 mAh cm^–2^, respectively, the areal loading and the information on porous structure for calendered 3D Cu-CNT, CE obtained from Li//3D-Cu-CNT-30 and -60 at a current density of 0.5 and 2.0 mA cm^–2^ with a total capacity of 0.5, 1.0, 2.0, and 4.0 mAh cm^–2^, the full cell test of 3D Cu CNT//LiFePO_4_ such as charge–discharge curves, cyclic voltammetry, and cycling performance and EIS (PDF)
