# Supplementary material for: 3D Porous Cu-Composites for Stable Li-Metal Battery Anodes
Source: ACS Nano. 2023 Jul 25;17(15):14658–66. doi: 10.1021/acsnano.3c02223 (PMC10416568; doi:10.1021/acsnano.3c02223)
Supplement: Supplementary file 1 — nn3c02223_si_001.pdf [file nn3c02223_si_001.pdf]

3D Porous Cu-Composites for Stable Li-Metal Battery Anodes

Sul Ki Park[a], *Davor Copic*[a,b], *Tommy Zijian Zhao*[a], *Agnieszka Rutkowska*[a], *Bo Wen*[a,c], *Kate Sanders*[a], *Ruhan He*[a], *Hyun-Kyung Kim*[d], *Michael De Volder*[*a]

[a] Department of Engineering, University of Cambridge, Cambridge, CB3 0FS, United Kingdom

[b] School of Engineering and Cyber Systems, United States Coast Guard Academy, New London 06320, United States

[c] Cambridge Graphene Centre, University of Cambridge, 9 JJ Thomson Avenue, Cambridge, CB3 0FA, United Kingdom

[d] Department of Materials Science and Engineering, Kangwon National University, Chuncheon 24341, Korea

[*] e-mail: mfld2@cam.ac.uk

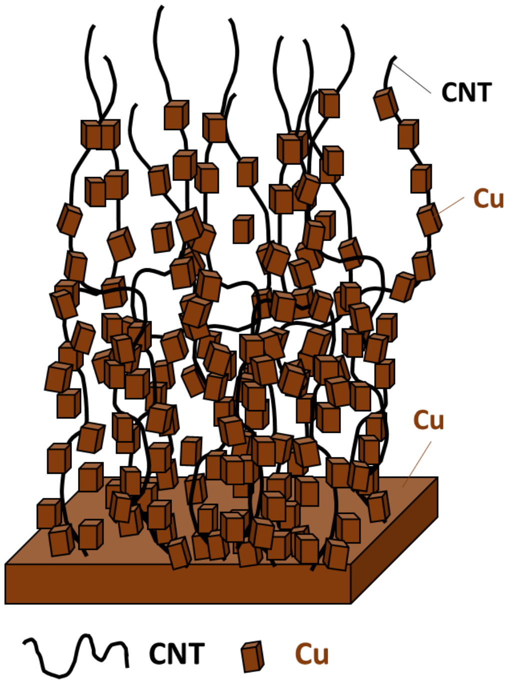

Figure S1. Schematic illustration of 3D porous Cu-CNT composite by the electrodeposition process.

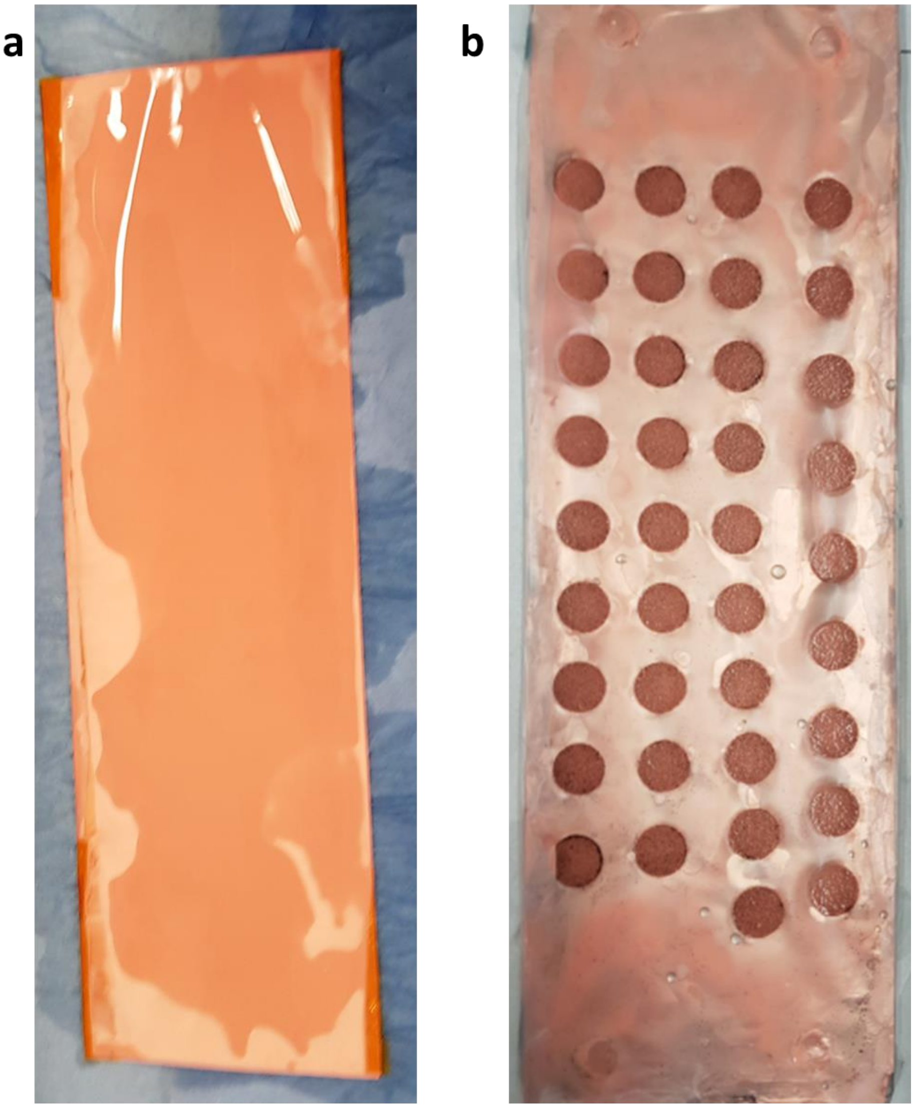

Figure S2. Pictures of (a) the Cu substrate before the electrodeposition and (b) Cu-CNTs plate on the Cu substrate after the electrodeposition.

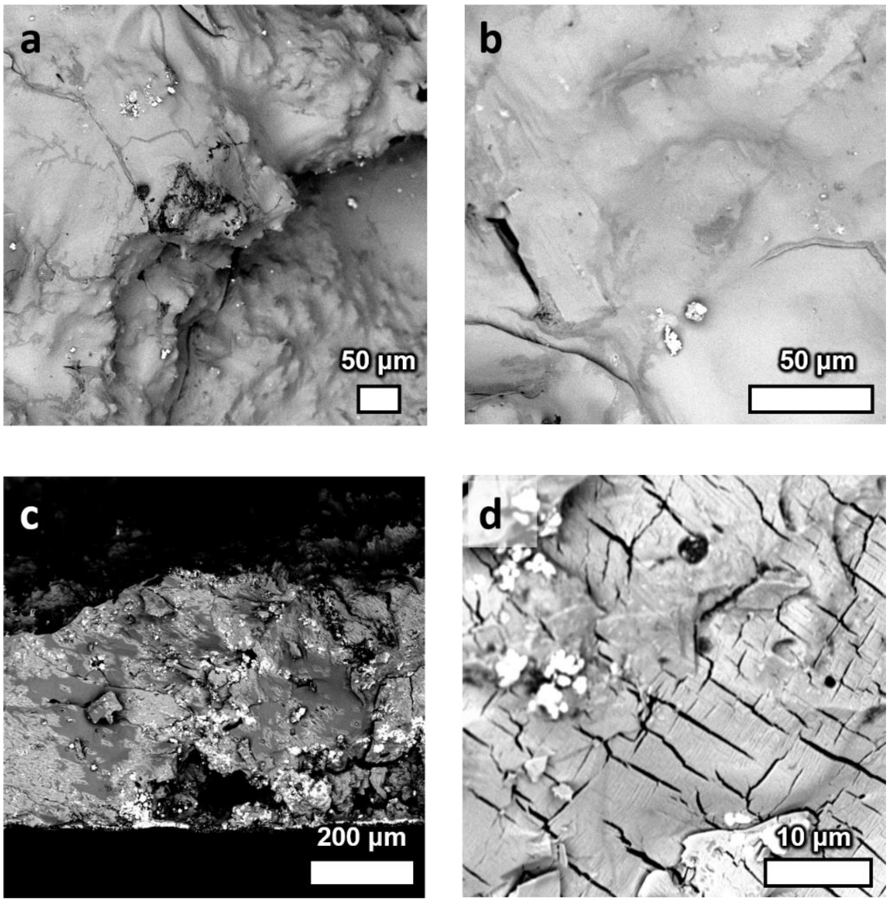

Figure S3. (a-b) Top and (c-d) cross-sectional SEM images of a Cu foam after the electrodeposition without CNTs.

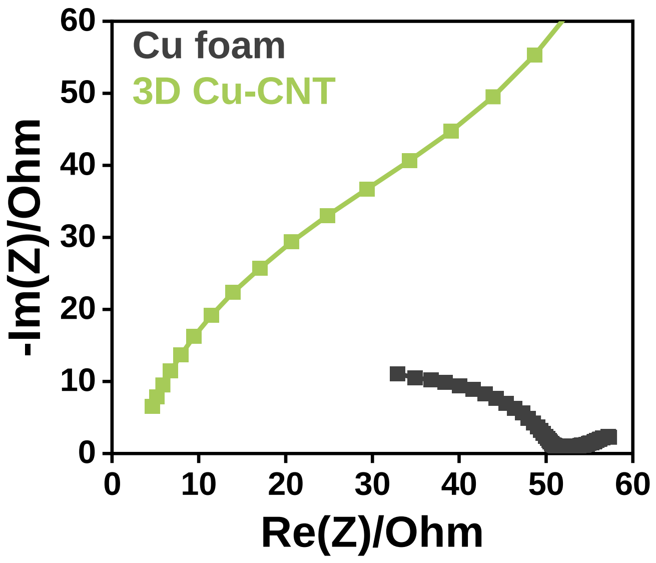

Figure S4. EIS of Cu foam and 3D Cu-CNT, which is with CNT and without CNT, respectively.

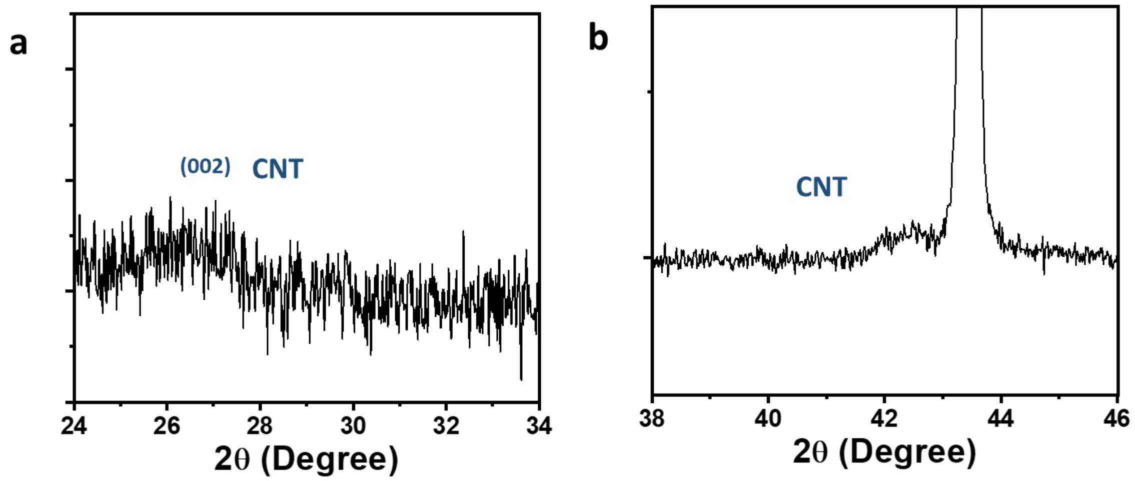

Figure S5. XRD pattern of 3D Cu-CNT.

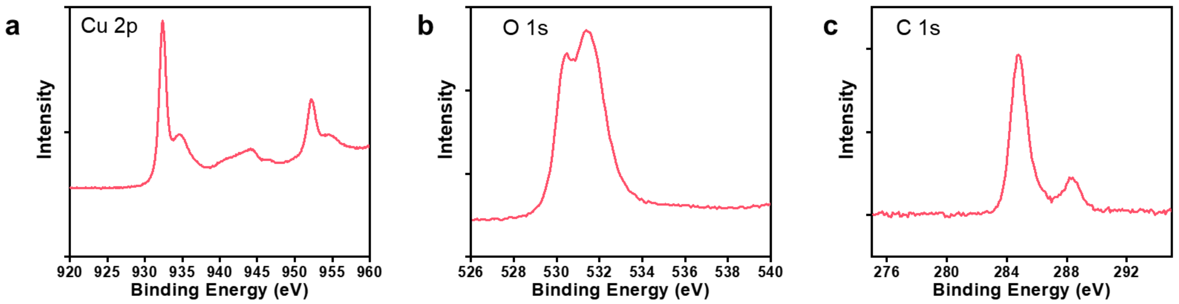

Figure S6. XPS spectra of (a) Cu 2p, (b) O 1s and (c) C 1s in 3D Cu-CNT.

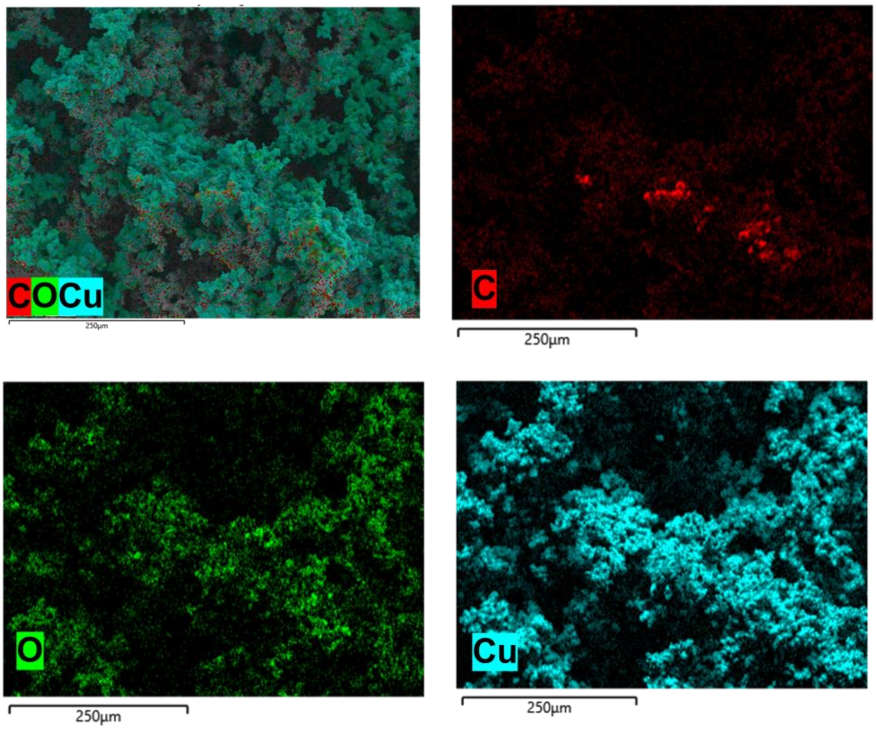

Figure S7. EDS mapping of 3D Cu-CNT.

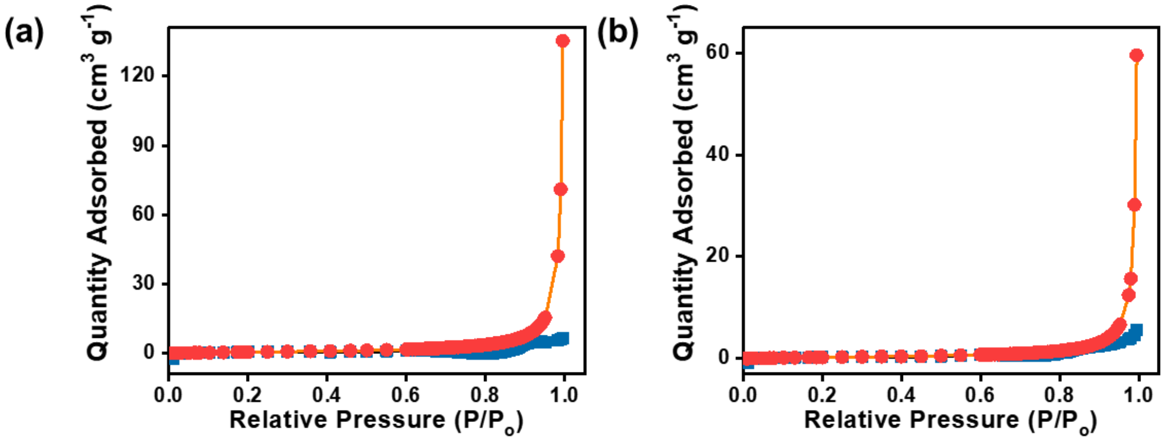

Figure S8. BET adsorption-desorption isotherm graph of (a) Cu-CNT-10 and (b)-30 after calendaring. BET data for 3D Cu-CNT-60 shows their curves are in the error range, which indicates its surface area can’t be estimated because of their too-low surface area per mass.

Table S1. The areal loading and the information of porous structure for 3D Cu-CNT-10, 30 and 60

**Electrodeposition time**	**10min**	**30min**	**60min**
Sample name	3D Cu-CNT-10	3D Cu-CNT-30	3D Cu-CNT-60
Area loading (g m^{-2})	20.19	40.55	99.02
Surface area(m^2 g^{-1})	3.39	1.39	~0
Pore volume (cm^3 g^{-1})	0.01	0.007	0.0005
Pore diameter by BJH adsorption (Å)	139	170	366

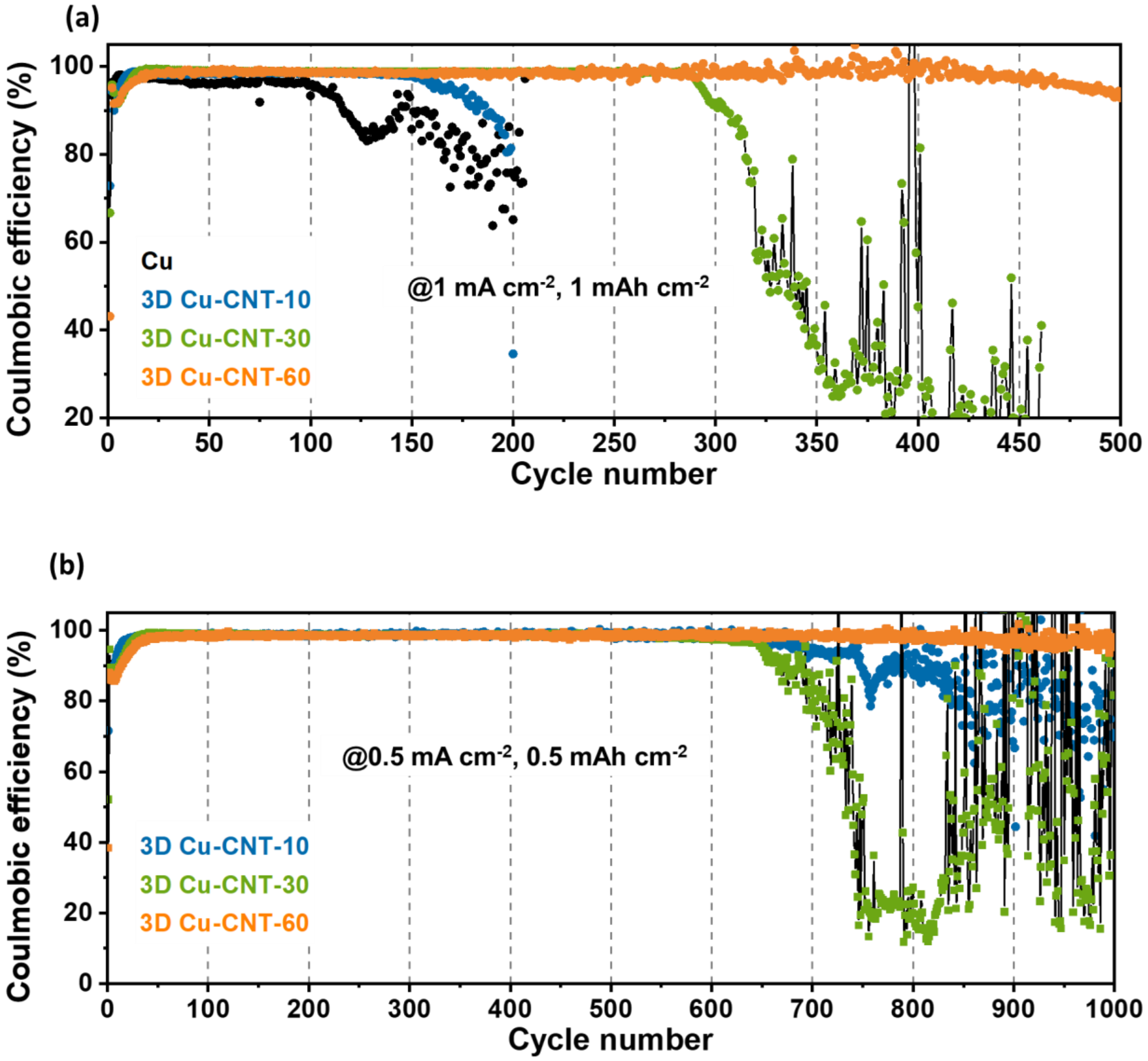

Figure S9. CE obtained from Li//Cu and Li//Cu-CNT-10, 30 and 60 at a current density of (a) 1 and (b) 0.5 mA cm^{-2} with a total capacity of 1 and 0.5 mAh cm^{-2}, respectively.

Table S2. The areal loading and the information of porous structure for 3D Cu-CNT-10, 30 and 60

	Before calendering				After calendering			
	Thickness (μm)	Density (g cm^{-3})	Li amount (mAh cm^{-3})	Stable Cycle (N)	Thickness (μm)	Density (g cm^{-3})	Li amount (mAh cm^{-3})	Stable Cycle (N)
Cu-CNT-10	82	1.58	121.95	350	54	2.40	185.19	180
Cu-CNT-30	255	1.31	39.22	530	86	3.88	116.28	330
Cu-CNT-60	486	1.89	20.58	785	150	6.12	66.67	230

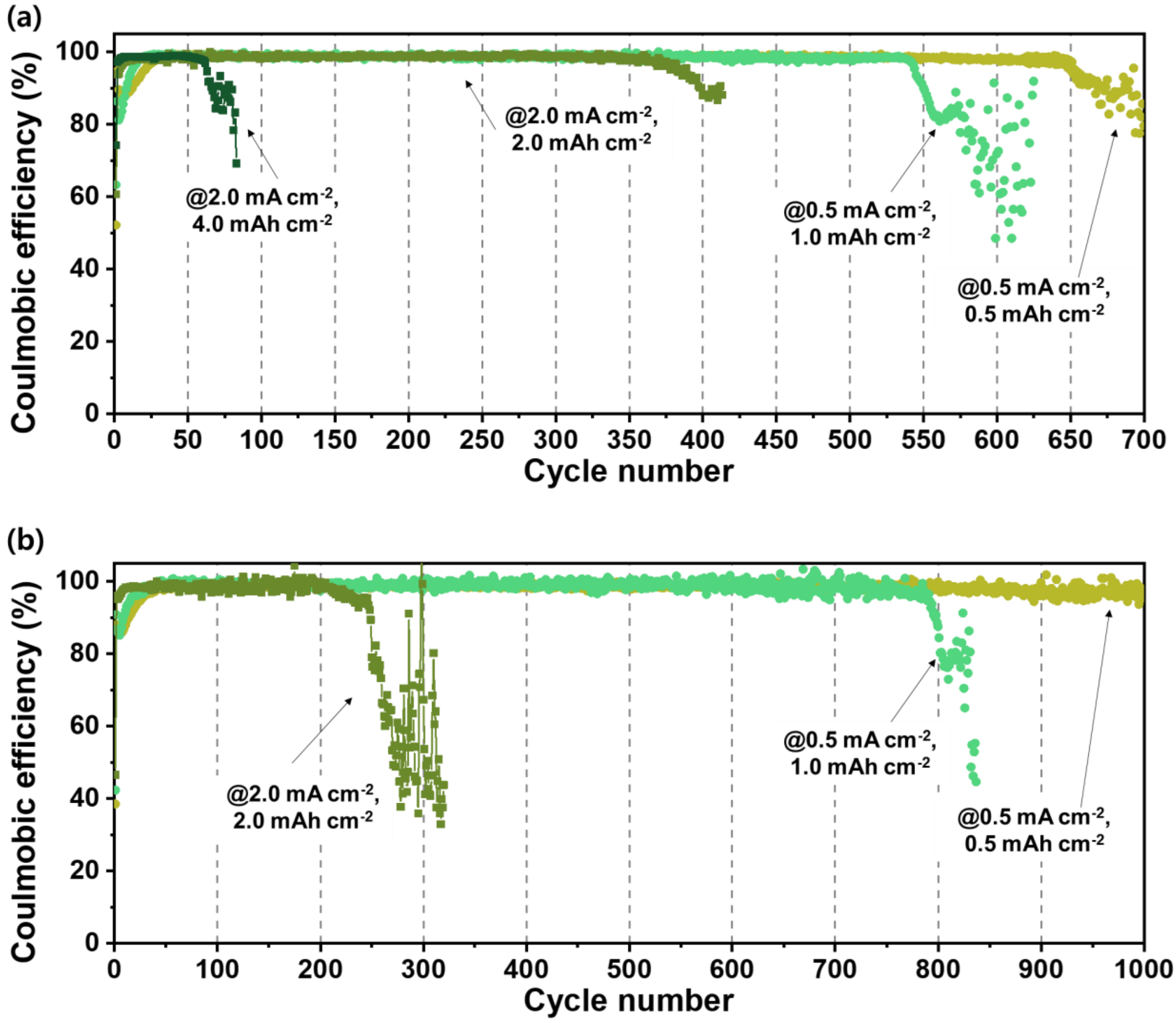

Figure S10. CE obtained from (a) Li//3D-Cu-CNT-30 and (b) -60 at a current density of 0.5 and 2.0 mA cm^{-2} with a total capacity of 0.5, 1.0, 2.0 and 4.0 mAh cm^{-2}

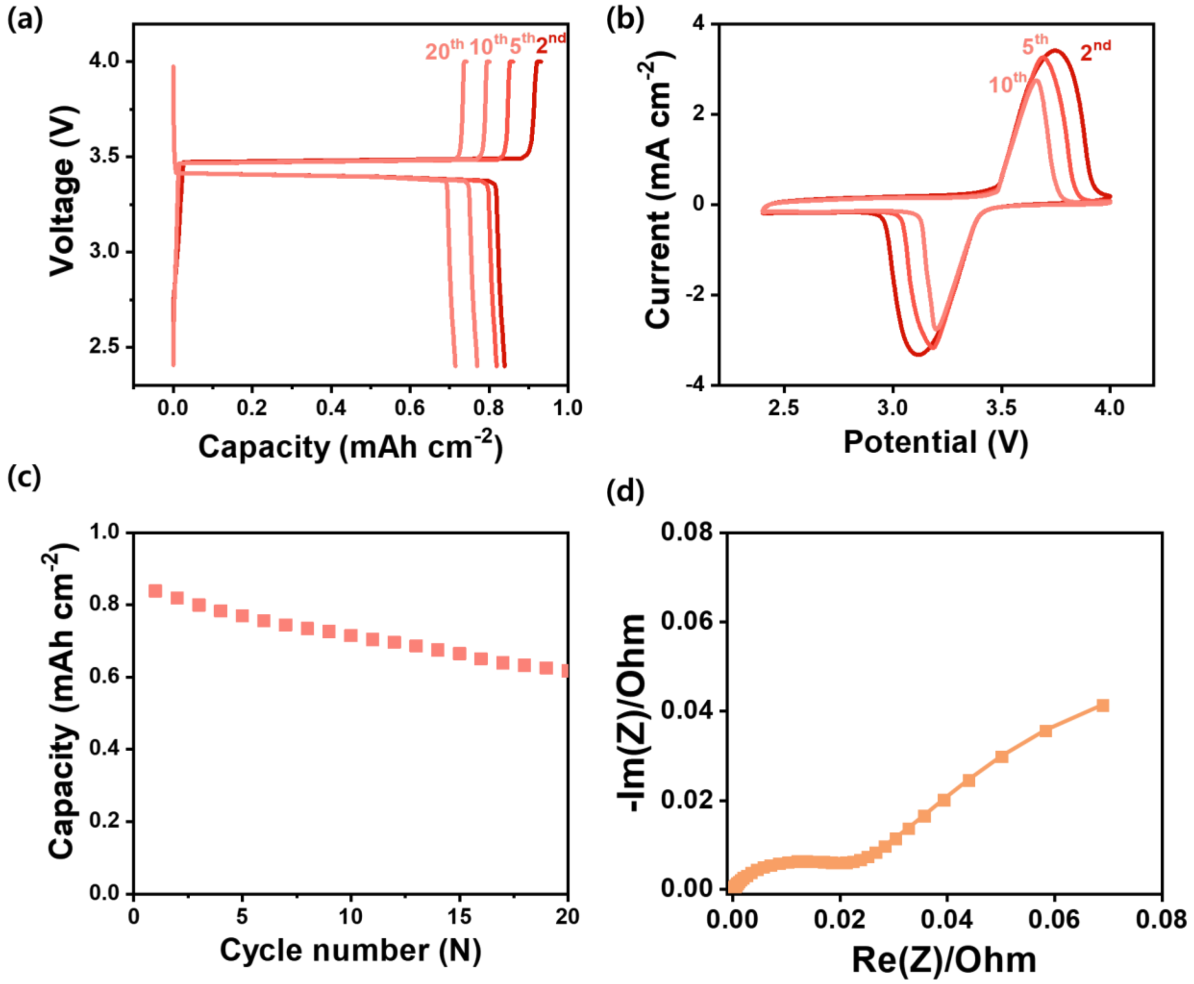

Figure S11. (a) 2nd, 5th, 10th and 20th charge-discharge curves, (b) 2nd, 5th and 10th cyclic voltammetry at 0.5 mV s^{-1}, (c) cycling performance at 0.2 C, and (d) Electrochemical Impedance Spectroscopy (EIS) from 10 kHz to 100 mHz of 3D Cu-CNT//$LiFePO_4$ (LFP) full cells.
